# The clinical efficacy of relocation pharyngoplasty to improve retropalatal circumferential narrowing in obstructive sleep apnea patients

**DOI:** 10.1038/s41598-020-58920-9

**Published:** 2020-02-07

**Authors:** Heonjeong Oh, Hyung Gu Kim, Suyeon Pyo, Jeong-Yeon Ji, Hyunjun Woo, Minju Kim, Dong-Young Kim, Chae-Seo Rhee, Hyun Jik Kim

**Affiliations:** Department of Otorhinolaryngology, Seoul National University College of Medicine, Seoul National University Hospital, Seoul, Korea

**Keywords:** Respiratory tract diseases, Outcomes research

## Abstract

Lateral pharyngeal wall appears to be a critical culprit of obstructive sleep apnea (OSA) subjects and relocation pharyngoplasty has been expected to be a promising surgical option to correct retropalatal circumferential narrowing in OSA patients. The purpose of our study is to evaluate the therapeutic outcomes of relocation pharyngoplasty and its clinical effectiveness in OSA patients with retropalatal circumferential narrowing. We performed relocation pharyngoplasty combined with nasal surgery in 133 OSA patients with the following characteristics: apnea-hypopnea index (AHI) over 10, retropalatal circumferential narrowing greater than grade I when awake, and redundant soft tissue around the lateral pharyngeal wall. The analysis of surgical success rate was performed with the data of 68 subjects who underwent pre and postoperative polysomnography. The objective success rate of relocation pharyngoplasty was 52.9%, and significant reduction of mean AHI with improvement of lowest SpO2 was seen in 69% of patients 3 months after the surgery. The median AHI was decreased from preoperative 37.3 to postoperative 21.4. Median lowest SpO2 changed from 78.4 to 84.1%. Total sleep time, daytime sleepiness, and visual analogue scale for snoring showed improvement as well. Postoperative complications including pain or bleeding were minimal in 133 subjects and a few patients complained of subtle taste loss. Our data demonstrate that relocation pharyngoplasty can be a favorable surgical option fighting against retropalatal circumferential narrowing.

## Introduction

Obstructive sleep apnea (OSA) is a common sleep disorder characterized by upper-airway collapse that causes reduction or cessation of airflow during sleep. Clinically, abnormal anatomy in the upper airway is obvious in OSA patients and reduced airflow from the nasal cavity and narrowing of the upper airway increase negative pressure in the pharyngeal airway and predispose the pharynx to collapse^1,2^. Both upper airway narrowing and increased airway resistance reportedly contribute to the underlying pathogenesis of OSA, leading to sleep-related symptoms such as loud snoring, apnea, fatigue, daytime sleepiness and systemic complications if not properly treated^3–8^. OSA occurs due to fixed or dynamic upper-airway obstruction caused by anatomical factors or abnormal upper-airway motor tone, and upper-airway obstruction can be caused by collapse at multiple levels, such as the soft palate, uvula, palatine tonsils, lateral pharyngeal walls, and base of the tongue^5,9^. A palatal pattern of collapse is most frequent and numerous surgical techniques have been designed to modify the palate anatomy in OSA patients^10^. Palatal surgeries for OSA aim to correct the pharyngeal tissues narrowing the upper airway, enhance the tension of the pharyngeal muscle, and widen the pharyngeal lumen. Multiple studies have demonstrated the clinical benefits of palatal surgeries, including relief from both subjective symptoms and life-threatening conditions in OSA patients, and the addition of new surgical options can potentially improve the success rate of management of OSA patients with palatal narrowing or collapse in their upper airway^11–15^.

The lateral pharyngeal wall is a complex structure composed of numerous pharyngeal muscle groups, such as the palatopharyngeus, superior pharyngeal constrictor, and palatoglossus muscles in addition to lymphoid tissue, including the palatine tonsils. Retropalatal circumferential narrowing due to lateral pharyngeal wall collapse has been documented to be a structural cause in the pathogenesis of OSA but its clinical significance has been underestimated in OSA patients undergoing palatal surgery^16^. The lateral pharyngeal wall is more collapsible or thicker in severe OSA patients than in normal subjects or patients with mild OSA, and retropalatal circumferential narrowing appears to be the sole independent risk factor in OSA patients^17^. Complete retropalatal circumferential narrowing may be closely related to higher apnea-hypopnea index (AHI) scores, and higher lateral pharyngeal collapsibility is seen in OSA patients who show a relapse of snoring or apneic events after surgery^16–20^. Surgical approaches that alter the retropalatal circumferential narrowing and maintenance of pharyngeal intensity to lateral dimensions appear to be critical for palatal surgeries in OSA patients with retropalatal circumferential narrowing. Therefore, diverse surgical techniques to reduce retropalatal circumferential narrowing and increase the tension and stability of the lateral pharyngeal wall have been introduced.

Lateral pharyngoplasty, expansion sphincter pharyngoplasty (ESP) and relocation pharyngoplasty have been introduced to correct retropalatal circumferential narrowing in OSA patients^21–24^. Relocation pharyngoplasty is most recently introduced surgical method to correct retropalatal circumferential narrowing and is assumed to be an effective technique for creating tension in the lateral pharyngeal walls with reduction of the number of apneic events or snoring intensity. In the present study, we aimed to determine the therapeutic outcomes of relocation pharyngoplasty in patients with mild, moderate, or severe OSA and investigate the clinical efficacy of relocation pharyngoplasty in OSA patients with retropalatal circumferential narrowing.

## Methods

### Ethics statement

One hundred thirty-three subjects diagnosed with OSA at the department of Otorhinolaryngology at Seoul National University Hospital from March 2015 to December 2018 were recruited, and retropalatal circumferential narrowing in these subjects was confirmed through drug-induced sleep endoscopy (DISE). Written informed consent was obtained from each participant and the study complied with the Declaration of Helsinki. The study protocol was approved by the Institutional Review Board of Seoul National University Hospital (IRB number1801-084-915).

### Patients and study design

All subjects underwent relocation pharyngoplasty, including tonsillectomy, a uvulopalatal (UP) flap procedure, and nasal surgeries to improve sleep-related symptoms and abnormal sleep parameters. Indications of relocation pharyngoplasty were (1) an AHI score greater than 10 events/hr on polysomnography (PSG), (2) retropalatal circumferential narrowing above DISE grade I (more than 50% narrowing), and (3) a narrowed oropharynx due to lateral pharyngeal collapse of the redundant soft tissue around the posterior pillar.

**Figure 1:**
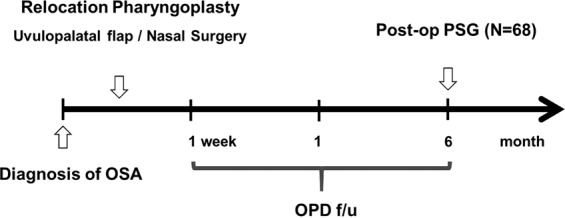
Schematic figure of the study design and clinical evaluation.

Patients’ medical records, including the results of pre- or postoperative PSG, were reviewed retrospectively. Total sleep time, AHI (events/hr), and lowest O_2_ saturation were observed before and at 6 months after the operation (Fig. 1). A successful procedure was defined as 50% reduction in AHI and an AHI < 20, as described by Sher^25^.

We also analyzed the therapeutic outcome of relocation pharyngoplasty with overall success and response rates which are defined commonly in the literature^26^. “Success” was defined as being AHI < 20 and ≥ 50% reduction in AHI and “improved” was defined as > 50% reduction in postoperative AHI.

Duration of hospital stay, early complications, primary outcome parameters such as the Epworth Sleeping Scale (ESS) and the visual analogue scale (VAS) for subjective snoring or apnea, and late complications were recorded at discharge from the hospital and during follow-up visits.

### Surgical technique for relocation pharyngoplasty

**Figure 2:**
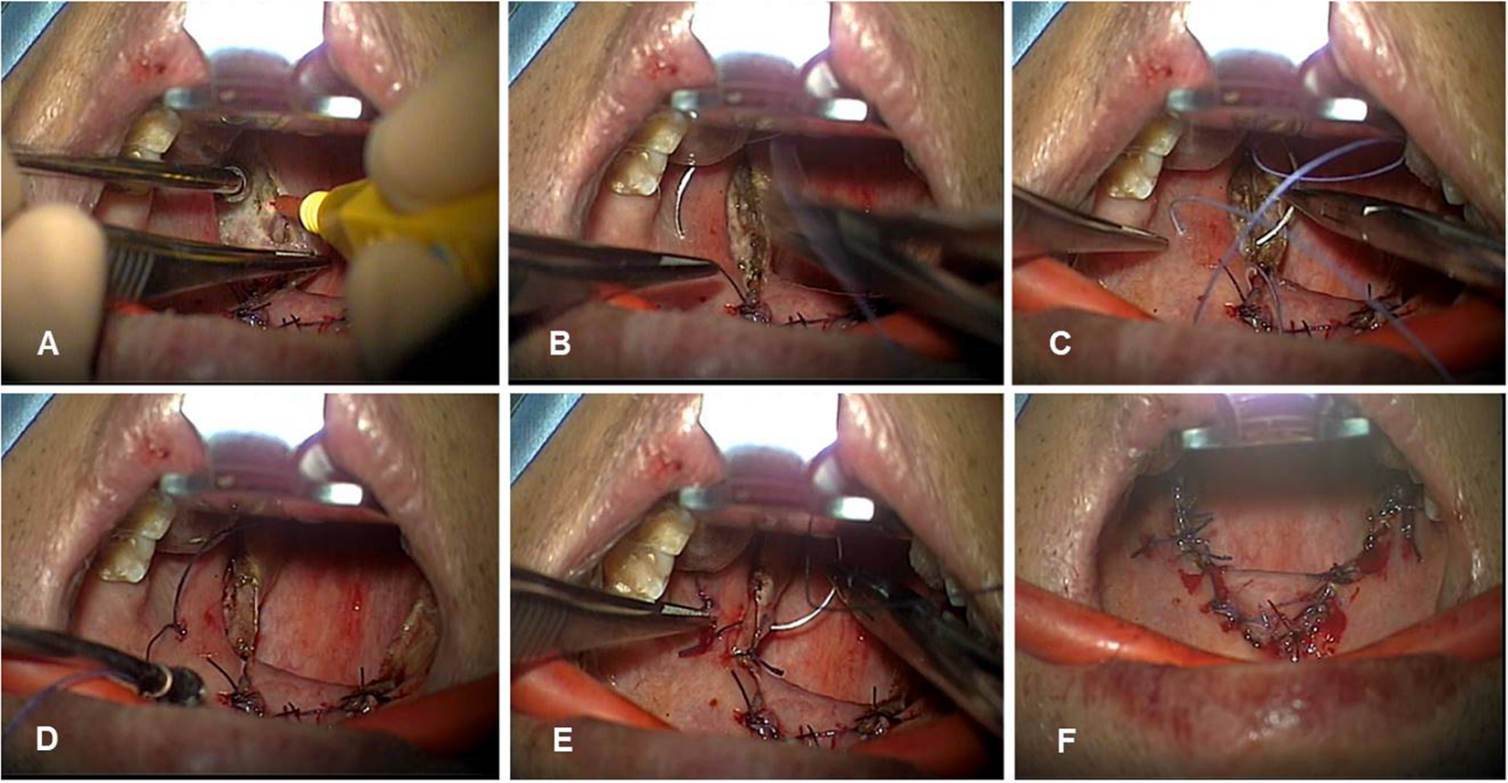
Main surgical steps of the relocation pharyngoplasty. (**A**) After completion of tonsillectomy, the palatopharyngeus muscle (*) is identified. (**B**) Palatopharyngeus muscle is dissected from posterior pillar including mucosa and submucosal adipose tissue. (**C**) The palatopharyngeus muscle is rotated cephalad and laterally to approach the anterior pillar. Absorbable suture is used to fix the palatopharyngeus muscle at the anterior pillar and two point sutures are performed with Vicryl #2.0. (**D**) Mucosal closure is carried out with Vicryl #3.0 and the same procedure is performed on the other side. (**E**) Relocation pharyngoplasty creates tension in the lateral pharyngeal wall.

**Figure 3:**
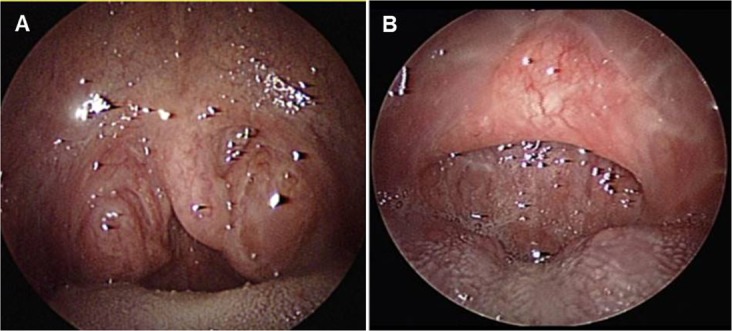
Operation findings prior to and following relocation pharyngoplasty.

The surgical technique of relocation pharyngoplasty in the current study was based on the method which was originally developed by Li *et al*.^18^. OSA subjects were selected for relocation pharyngoplasty depending on the presence of oropharyngeal collapse, as determined by a complete preoperative upper airway examination and DISE using the VOTE classification, which is determined by anatomical analysis of the velum, oropharynx, tongue base and epiglottis^27^. The patients did not present with obstruction at the tongue base or epiglottis. All subjects complained of nasal obstruction, and were scheduled to receive septoturbinoplasty to obtain improved nasal airflow. The surgery was performed under general anesthesia with the patient in the Rose position and nasotracheal intubation. Bilateral tonsillectomy and the UP flap procedure were performed first. After indicating the design with a marking pen on the soft palate above the uvula, mucosa was removed with sharp pointed scissors or Bovie cautery and only uvular mucosa was carefully removed to prevent damage to the uvular muscle. The uvular tip was then turned over onto the soft palate, and the abundant uvula tip was cut properly with Bovie cautery. The palatoglossus (PG), palatopharyngeus (PP), and superior pharyngeal constrictor (SPC) muscles were identified following the UP flap procedure. The PP muscle was divided from the SPC muscle and posterior pillar mucosa to facilitate tension-free approximation. The PP muscle near the uvula was then rotated cephalad and laterally to approach the counterpart of the soft palate. The SPC muscle was grasped and sutured laterally to the ipsilateral PG muscle in a cephalic-to-caudal direction with Vicryl #2.0 (Ethicon, Somerville, NJ) through a mattress-style suture (Fig. 2). The suture usually required two or three separated stitches to splint the SPC muscle in one tonsillar fossa. Mucosal closure between the anterior and posterior pillars used Vicryl #3.0. The tension of the suture on the flap was adjusted to prevent postoperative wound dehiscence. The same procedure was repeated on the opposite side (Fig. 3). All subjects were discharged 2 days after relocation pharyngoplasty, and the last follow-up visit was performed 3 months after the surgery.

### Statistical analysis

Statistical calculations were performed using SPSS 19.0 (SPSS, IBM). Postsurgical changes in AHI, lowest oxygen saturation, ESS value, and VAS for snoring or apnea were analyzed using a paired t-test and the Wilcoxon signed-rank test. Descriptive data are presented as mean ± standard deviation. Differences are considered significant if *p* < 0.05.

## Results

### Demographic data of subjects

We recruited 133 subjects who were diagnosed with OSA and underwent relocation pharyngoplasty to resolve airway narrowing and reduce retropalatal circumferential narrowing. Of them, 117 patients were men, and 16 were women. The mean age was 43.4 years (range, 21–56 years), and the mean body mass index was 26.6 kg/m^2^ (range, 16–34.2 kg/m^2^). The severity of OSA was based on AHI: 23 patients demonstrated mild OSA, 52 showed moderate OSA, and 58 showed severe OSA. We did not recommend relocation pharyngoplasty to OSA patients with an AHI below 10 events/hr even if they showed lateral pharyngeal collapse on DISE. We performed relocation pharyngoplasty in OSA patients with grade I or larger tonsils and 78.9% of subjects exhibited grade II or higher tonsil grade. In addition, 81.9% of subjects showed a palatal grade above II. Based on preoperative DISE findings, all subjects showed circumferential narrowing at the retropalatal level and the mean grade of retropalatal narrowing was 2.3 ± 0.7 (Table 1). Sixty-eight subjects had PSG prior to and after relocation pharyngoplasty and preoperative PSG findings revealed that the mean AHI was 35.0 ± 20.7 events/hr, mean lowest O_2_ saturation was 77.9 ± 12.1%, mean total sleep time was 393.7 min, and mean REM sleep percentage was 21.6%.

**Table 1:** Desmographic details of subjects (N = 133).

Mean Age (SD),	43.7 (13.7)
Male	89.3% (N = 117)
Female	12.2% (N = 16)
Mean BMI, (SD)	26.6 (3.33)
Obese	71.8% (N = 94)
**Tonsil grade**	
I	21.1% (N = 28)
II	31.6% (N = 42)
III	38.3% (N = 51)
IV	9.0% (N = 12)
**Palatal grade**	
I	18.1% (N = 24)
II	28.7% (N = 38)
III	32.3% (N = 43)
IV	21.1% (N = 28)
**Mean DISE narrowing grade (SD)**	
Retropalatal circumferential	2.3 (0.70)
Retroglossal	1.3 (0.84)

SD: standard deviation, DISE: Drug-induced sleep endoscopy.

### Therapeutic outcome of relocation pharyngoplasty

**Figure 4:**
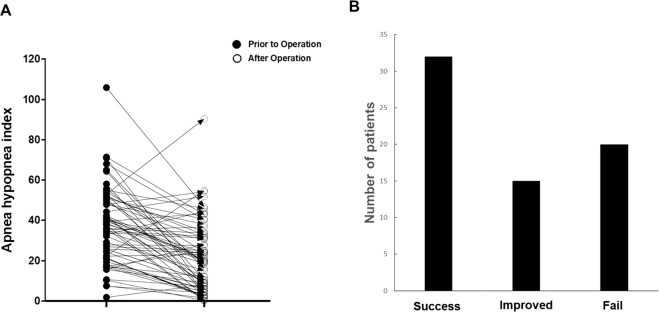
Changes in sleep parameters of OSA subjects following relocation pharyngoplasty. (**A**) Apnea and hypopnea index (*p < 0.05 when comparing grades between OSA subjects before and after relocation pharyngoplasty). (**B**) The overall success and response rates, with “success” being AHI < 20 and ≥ 50% reduction in AHI, and “improved” being postoperative AHI ≥ 20 and reduction > 50%, “failed” being AHI reduction under <20%.

The therapeutic outcome was determined using AHI scores from PSG, which was performed at 6 months after operation (N = 68). The mean AHI for entire subjects was 35.0 ± 20.7 events/hr prior to relocation pharyngoplasty, which decreased significantly to 21.4 ± 17.6 postoperatively. Selecting an arbitrary threshold of a 50% reduction in AHI and an AHI < 20, the number of responders was 36, for a success rate of sleep surgery (including relocation pharyngoplasty) of 52.9% in OSA patients with retropalatal circumferential narrowing (Fig. 4A). Overall success and response rates are analyzed with “success” being a  ≥50% reduction in AHI and a postoperative AHI < 20, and “improved” being a  ≥ 50% reduction in AHI^26^. This resulted in 69.1% of patients being assigned to the success and improved  group (Fig. 4B). In particular, moderate OSA patients showed higher successful outcomes following relocation pharyngoplasty (68.2%) compared with mild or severe OSA patients with retropalatal circumferential narrowing.

As a next step, we classified these 68 subjects depending on tonsil grade and compared therapeutic outcome of relocation pharyngoplasty according to tonsil size of subjects with an arbitrary threshold of a 50% reduction in AHI and an AHI < 20. The results showed that success rate of relocation pharyngoplasty in subjects with grade I tonsils (N = 28) was 35.7%, in subjects with grade II (N = 25) was 64.0%, in subjects with grade III (N = 11) was 54.5%, and in subjects with grade IV (N = 4) was 100%. We found that success rate of sleep surgeries including relocation pharyngoplasty was relatively higher in OSA patients with grade II, III, and IV compared to the OSA subjects with grade I tonsils.

Postoperative PSG results also showed that the lowest O_2_ saturation improved significantly from 78.46 ± 9.5% to 84.08 ± 6.4%. In addition, postoperative total sleep time was prolonged from 390.2 ± 69.4 to 411.9 ± 54.7 minutes. In the subjective symptoms, the mean ESS value improved from 8.77 ± 3.7 to 5.76 ± 3.3, and the sleep efficiency and REM sleep percentage improved significantly in 68 subjects after surgery (Table 2).

**Table 2:** Comparison of baseline and postoperative subjective symptoms and polysomnographic parameters in OSA patients with lateral pharyngeal wall narrowing (N = 68).

	Pre-op [mean (SD)]	Post-op [mean (SD)]	Difference [mean (SD)]	p-value
AHI	37.32 (18.68)	21.41 (17.61)	15.91 (19.52)	<0.0001
Apnea index	17.27 (16.91)	5.333 (7.431)	11.93 (17.70)	<0.0001
Min SpO_2_ (%)	78.46 (9.560)	84.08 (6.360)	5.617 (8.208)	<0.0001
Total sleep time (min)	390.2 (69.38)	411.9 (54.74)	21.77 (79.73)	0.034
Snoring	moderate to severe	mild to moderate	—	<0.0001
ESS	8.771 (3.775)	5.769 (3.270)	1.667 (1.528)	0.1994
Sleep efficiency (%)	86.64 (8.387)	88.52 (6.903)	1.881 (9.555)	0.1578
REM percentage (%)	21.85 (6.929)	22.16 (5.733)	0.3115 (7.493)	0.8338

Op: operation, SD: standard deviation, AHI: apnea hypopnea index, Min SpO_2_: minimal oxygen saturation, ESS: Epworth sleepiness scale, REM: rapid eye movement.

Mucosal wound dehiscence was observed in 12.7% (N = 133) of subjects after relocation pharyngoplasty combined with the UP flap procedure. Partial dehiscence was observed in 11 subjects, and total dehiscence was observed in 6. We did not suture mucosal dehiscence, and the occurrence of dehiscence was not correlated with the failure rate of relocation pharyngoplasty. The mean admission period was 4.01 ± 0.4 days, and tracheostomy was not performed in any subjects following relocation pharyngoplasty.

**Figure 5:**
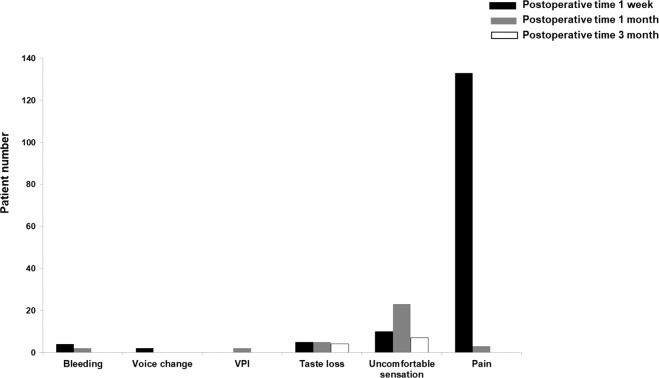
Subjective complaints or complications following relocation pharyngoplasty. Subjective symptoms: postoperative pain, uncomfortable sensation, taste, VPI, voice change, and bleeding were investigated 1 week, 1 month, and 3 months after relocation pharyngoplasty (VPI: velopharyngeal insufficiency).

We investigated complications or side effects following relocation pharyngoplasty: postoperative pain, uncomfortable sensation, taste loss, velopharyngeal insufficiency, voice change, and postoperative bleeding (Fig. 5). We did not observe serious complications related to relocation pharyngoplasty at 1 week, 1 month, or 3 months after surgery. In total, all 133 subjects complained of pain at 1 week after relocation pharyngoplasty, but only 4 patients still experienced oropharyngeal pain at 1 month after relocation pharyngoplasty. No patient experienced postoperative pain at 3 months after relocation pharyngoplasty. In addition, 24 patients felt an abnormal sensation around the soft palate area 1 week after surgery, but only 4 patients complained of it at 3 months postoperatively. Some patients complained of velopharyngeal insufficiency, voice change and taste loss after relocation pharyngoplasty, but those complaints had subsided by at 1 month. We observed postoperative bleeding in 5 patients at the oropharynx after relocation pharyngoplasty and postoperative bleeding was well controlled in all patients through conservative management without the need for electrocautery or bleeder ligation.

## Discussion

Through this study, we found that relocation pharyngoplasty can be an adequate surgical option to improve upper airway narrowing at the retropalatal level and provide positive therapeutic outcomes for OSA patients with retropalatal circumferential narrowing in the upper airway. Our current clinical results also suggest possible surgical indications for relocation pharyngoplasty in OSA patients: PSG results, DISE, and endoscopic findings such as a narrowed oropharynx due to tonsil enlargement (>grade I), lateral bulk of soft tissue around the posterior pillar, and circumferential narrowing (DISE grade > I) at the retropalatal level.

Lateral pharyngeal wall collapse contributes to the pathogenesis of OSA by increasing airway resistance and causing partial or complete obstruction of the upper airway^28–30^. Retropalatal circumferential narrowing due to lateral pharyngeal wall collapse is more closely related to airway resistance and causes aggravated hypoxic tissue damage in OSA patients^30^. In OSA patients, the lateral pharyngeal wall is more collapsible when pressured by airflow than in healthy subjects and the lateral pharyngeal wall of OSA patients can be thicker than in normal subjects, making it a predominant anatomic factor inducing upper-airway collapse in OSA patients^19,29^. In addition, OSA patients with retropalatal circumferential narrowing exhibited higher AHI and respiratory disturbance index scores than OSA patients with anteroposterior upper airway narrowing^31^. This suggests a need for an adequate surgical option to reduce retropalatal circumferential narrowing in OSA patients and adequate surgical reduction of retropalatal circumferential narrowing provide a satisfactory therapeutic outcome in OSA patients. Therefore, accurate evaluation of retropalatal circumferential narrowing and the maintenance of tension or stability of the lateral pharyngeal wall may be critical in sleep surgeries for OSA patients who show circumferential narrowing of upper airway.

Uvulopalatopharyngoplasty (UPPP) is a popular technique in the field of sleep surgery. However, because conventional UPPP widens the retropalatal space from anterior to posterior, it appears to be ineffective in OSA patients with multiple-level obstructions, and therapeutic outcomes of UPPP are unsatisfactory in OSA patients with retropalatal circumferential narrowing^32,33^. Diverse surgical techniques to replace UPPP have been introduced to correct lateral pharyngeal wall collapse in OSA subjects. Lateral pharyngoplasty, relocation pharyngoplasty, and ESP have been suggested as effective surgical procedures to correct retropalatal circumferential narrowing. These surgical techniques are associated with better improvement of AHI scores than UPPP and more effectively widen the pharyngeal lumen by reducing collapse of the lateral pharyngeal wall during sleep (Cahali., 2003; Li and Lee., 2009; Pang and Woodson., 2007; Woodson *et al*., 2012; Cahali *et al*., 2004)^21–24,34^. We usually recommend relocation pharyngoplasty and ESP to OSA patients with retropalatal circumferential narrowing to improve lateral pharyngeal wall tension. Although lateral pharyngoplasty produced better clinical and PSG outcomes in OSA patients than did UPPP, cross-sectional measurements of the pharyngeal airway did not differ between treatments^16^. In addition, many patients experienced significant dysphagia following lateral pharyngoplasty because it is an invasive process that undermines the superior pharyngeal constrictor muscle, and lateral pharyngoplasty exhibited more complications, such as oronasal reflux of liquid and wound dehiscence, than did relocation pharyngoplasty and ESP^14,21^.

Relocation pharyngoplasty appears to be similar to lateral pharyngoplasty in splinting the lateral pharyngeal wall but has the distinctive characteristic of plicating the superior pharyngeal constrictor muscle to prevent scarring contraction of the tonsillar fossa^22^. Advancement of the soft palate is achieved by removing the supratonsillar mucosa and adipose tissue to create a space for reconstruction of the velopharynx^22^. In addition, this procedure preserves the palatal muscle by removing only part of the soft palate mucosal layer. Our study included patients with mild, moderate, or severe OSA who showed more than 50% narrowing in their lateral pharyngeal wall with tonsil enlargement, and we expected them to need more tension to improve lateral pharyngeal wall collapse. Based on our results, relocation pharyngoplasty resulted in approximately 53% successful surgical results in OSA patients and provided a higher success rate in moderate OSA patients, with 69% of OSA patients showing an improved AHI score and superior numerical changes in sleep parameters following relocation pharyngoplasty. We conclude that relocation pharyngoplasty is a favorable surgical option that maintains pharyngeal function in moderate OSA patients with retropalatal circumferential narrowing.

It has been reported that relocation pharyngoplasty has obvious advantages in splinting the lateral pharyngeal wall. Relocation pharyngoplasty does not undermine the SPC muscle and avoids the possibility of damaging manor neurovascular structures. Our data showed that OSA patients who underwent relocation pharyngoplasty complained of minimal complications and most complications were resolved within 1 month of relocation pharyngoplasty.

Maintenance of muscle tension after pharyngeal suture in relocation pharyngoplasty may be critical for successful surgical outcomes in OSA patients with lateral pharyngeal wall collapse. We presume that the tension that relocation pharyngoplasty provides in the lateral pharyngeal wall is not as powerful as that provided by ESP, which repositions the underlying muscular structures of the pharynx and palate to widen the lateral pharyngeal airway. After revealing the arching fibers of the palate muscles, isolated PP muscle is attached to the arching fiber of the palate muscle^23^. ESP thus creates more powerful tension in the lateral pharyngeal wall and reduces the bulk of lateral pharyngeal soft tissue by isolating and rotating the PP muscle super-anterolaterally. In a previous study, we found ESP combined with uvuloplasty to be an effective surgical option for lateral pharyngeal collapse in patients with severe OSA with minimal complications^14^. Based on our clinical data, relocation pharyngoplasty also provided a high cure rate for OSA patients with lateral pharyngeal collapse and effectively reduced lateral pharyngeal bulk, resulting in enhanced soft-palate tension or widening of the pharynx without severe complications after surgery. Furthermore, subjective symptoms related to OSA, such as snoring, apnea, and daytime sleepiness, also improved significantly after relocation pharyngoplasty. The lowest oxygen saturation and valid sleep times also improved, and the extent of the retropalatal area in the upper airway widened significantly after relocation pharyngoplasty. We therefore estimated that relocation pharyngoplasty may be more useful for moderate OSA patients with greater than grade I circumferential narrowing at the retropalatal level and greater than grade I tonsil enlargement. However, we still suggest ESP for OSA patients who exhibit more severe lateral pharyngeal collapse of grade II or higher and a narrowed oropharynx due to bulky soft tissue in the lateral pharyngeal wall.

In summary, our clinical findings confirm that relocation pharyngoplasty is a useful surgical option in OSA subjects with retropalatal circumferential narrowing due to lateral pharyngeal wall collapse. They also supply evidence supporting the favorable indications of relocation pharyngoplasty and provide improved therapeutic outcomes for patients with moderate OSA and relatively less severe retropalatal circumferential narrowing.
